# In ovarian cancer the prognostic influence of *HER2/neu* is not dependent on the *CXCR4*/*SDF-1* signalling pathway

**DOI:** 10.1038/sj.bjc.6603581

**Published:** 2007-01-23

**Authors:** D Pils, A Pinter, J Reibenwein, A Alfanz, P Horak, B C Schmid, L Hefler, R Horvat, A Reinthaller, R Zeillinger, M Krainer

**Affiliations:** 1 Department of Internal Medicine I, Division of Oncology, Medical University of Vienna, Waehringer Guertel 18-20, Vienna A-1090, Austria; 2 Department of Obstetrics and Gynecology, Division of Gynecology, Medical University of Vienna, Vienna 1090, Austria; 3 Clinical Pathology, Medical University of Vienna, Vienna 1090, Austria

**Keywords:** ovarian cancer, immunohistochemistry, tissue microarray, *HER2/neu*, *CXCR4*, *SDF-1*

## Abstract

*HER2/neu* overexpression is a driving force in the carcinogenesis of several human cancers. In breast cancer the prognostic influence of *HER2/neu* was shown to be at least partly based on increased metastatic potential mediated by the chemokine–chemokine receptor pair *SDF-1(CXCL12)/CXCR4*. We wanted to evaluate the influence of *HER2/neu* on ovarian cancer prognosis and to investigate whether compromised survival would correlate with *CXCR4* expression and/or *SDF-1* abundance. Therefore, we analysed *HER2/neu*, *CXCR4*, and *SDF-1* in 148 ovarian tumour samples by means of immunohistochemistry on tissue microarrays. Overexpression of *HER2/neu* was found in 27.6% of ovarian cancer tissues and in 15% of ovarian borderline tumours. In ovarian cancer patients, overexpression of *HER2/neu* correlated closely with overall survival (univariate hazard ratio (HR) 2.59, *P*=0.005; multiple corrected HR 1.92, *P*=0.074). In contrast, *CXCR4* expression and *SDF-1* abundance had no impact on overall survival, and both parameters were not correlated with *HER2/neu* expression. As expected, cytoplasmic *CXCR4* expression and *SDF-1* abundance correlated closely (*P*<0.0001). Our results confirm a univariate influence of *HER2/neu* expression on overall survival, which was completely independent of the expression of *CXCR4* and the abundance of *SDF-1*, implying significant differences between the *HER2/neu* downstream pathways in ovarian cancer compared with breast cancer.

Epithelial ovarian cancer is the most lethal gynaecologic malignancy and with about 6% the fourth most frequent cause of cancer-related death of women in Western countries. Estimates indicate that one in 70 women will develop ovarian cancer in her lifetime, with a median survival rate of 4.5 years. A recent cancer statistic reported an estimated 22 200 new cases and 16 210 deaths per year in the United States (Jemal *et al*, 2005). Early diagnosis is a major challenge, as more than three-quarter of cases are diagnosed in late stages. Ovarian cancer metastasises preferentially to the local lymph nodes and the peritoneum, and in contrast to breast cancer, only rarely in other organs like liver, lung, and bones.

The majority of hereditary ovarian cancer cases are caused by germline mutations in *BRCA1* and *BRCA2*, the so-called breast/ovarian cancer syndrome. Mutations in these caretaker genes set the scene for further genomic and epigenomic aberrations, which ultimately transform healthy cells into cells with perturbed cell cycle control and metastatic potential. The starting point of sporadic cases may differ but the sequence of events is not well described to date, for ovarian cancer in particular.

Ovarian cancer and breast cancer share the overexpression of *HER2/neu*, a member of the HER family of receptor tyrosine kinases triggering signalling pathways which control cell growth, differentiation, motility, and adhesion. In breast cancer, the prognostic value of *HER2/neu* expression is well established and the therapeutic modulation of this oncogene by antibodies or small molecules is a classic example of targeted therapy. The situation in ovarian cancer is less clear (recently reviewed in Serrano-Olvera *et al*, 2006), with contradicting results of *HER2/neu* expression in the prognosis of the disease and very little available data on its potential for therapeutic manipulations, prompting us to investigate *HER2/neu* expression and its potential consequences in ovarian cancer.

For breast cancer, it was shown that *HER2/neu* mediated tumour metastasis, and survival prognosis is essentially driven by upregulation of the chemokine receptor *CXCR4*, a membrane-bound G-protein-coupled receptor (Li *et al*, 2004). The chemokine was first described in its function as a key regulator of the homing process of lymphocytes to inflammatory tissues (Bleul *et al*, 1997). In previous reports dealing with *CXCR4* expression in neoplastic diseases nuclear, cytoplasmic, and membrane staining was found by means of immunohistochemistry. Recently, for breast cancer, it was shown that only cytoplasmic staining of *CXCR4* had significant impact on prognosis, but not nuclear staining – using the same antibody as we used for our study (Salvucci *et al*, 2005). Three previous studies identified nuclear localisation of *CXCR4* in hepatocellular carcinoma (Shibuta *et al*, 2002), invasive ductal mammary carcinoma (Kato *et al*, 2003), and non-small-cell lung cancer (NSCLC) (Spano *et al*, 2004). Strong *CXCR4* nuclear staining was associated with significantly better outcome in early-stage NSCLC (Spano *et al*, 2004). The natural ligand of *CXCR4*, the stromal cell-derived factor (*SDF-1* or *CXCL12*), is highly expressed in lung, liver, and lymph nodes (Phillips *et al*, 2003), the preferred organs for metastasis of several tumours.

To contribute to the controversial discussion about the influence of *HER2/neu* overexpression on ovarian cancer prognosis and whether the chemokine receptor system *SDF-1*/*CXCR4* is significantly involved in this process, we examined the expression of these three potential oncoproteins by means of immunohistochemistry on tissue microarrays comprising 148 ovarian cancer patients.

## MATERIALS AND METHODS

### Ovarian tissue microarray and immunohistochemistry

For immunohistochemical studies, paraffin material available from primary diagnosis was used. Patients gave informed consent according to the criteria of the Medical University of Vienna. Relevant clinical information was collected and tissue samples and clinical data anonymised. A tissue microarray was composed by taking core needle ‘biopsies’ from specific locations in the preexisting paraffin-embedded tissue blocks and re-embedding them in an arrayed master block, using techniques and an apparatus developed by Beecher Instruments Inc., Micro-Array Technology (Sun Prairie, WI, USA). To achieve good representation of the tumour, three biopsies of tumour material were selected from each patient. Using this technology, each tissue sample was treated in an identical manner and the entire cohort was analysed in one batch on three slides. Reagent conditions, incubation times and temperatures, wash conditions, and antigen retrieval (if necessary) were held identical for each case. A 4–5 *μ*m paraffin section of the tissue microarray was deparaffinised (xylene) and rehydrated (incubation in serial dilutions of ethanol), and, subsequently, the sections were treated with 0.2% H_2_O_2_/PBS (pH 7.4) to quench endogenous peroxidases. After blocking with 2% normal serum (from the animal in which the secondary antibody was raised) for 30 min, the sections were incubated at 4°C overnight with primary antibodies (*CXCR4*, mouse monoclonal anti-human *CXCR4* (MAB172) (R&D Systems, Minneapolis, MN, USA); *SDF-1*, mouse monoclonal anti-human/mouse *SDF-1*/*CXCL12* antibody (MAB350) (R&D Systems); *HER2/neu* (DAKO, Glostrup, Denmark); and HercepTest (DAKO, Glostrup, Denmark). As secondary antibody for *CXCR4* and *SDF-1*, an anti-mouse Ig, horseradish peroxidase-linked whole antibody from sheep (NA 931, Amersham, Buckinghamshire, UK) was utilised. Staining was performed using a staining kit from DAKO: DAKO Cytomation Liquid DAB+substrate (Glostrup, Denmark). DAKO HercepTest was carried out and stained as described by the manufacturer. Positive and negative control slides (as appropriate) were stained within the same batch with the tissue microarrays and examined before evaluation of the tissue microarrays.

### Data analyses and statistics

Staining of the tissue microarrays was interpreted by two independent pathologists. Classification of all three tissue microarrays was performed at once to ensure reliability and reproducibility. Staining for *CXCR4* was classified in ‘1’ (missing or very low cytoplasmic expression), ‘2’ (medium cytoplasmic expression), and ‘3’ (high cytoplasmic expression), and in addition ‘−’ (no) for negative nuclear staining or ‘+’ (yes) for positive nuclear staining. Staining of *SDF-1* was classified in ‘1’ (missing or very low membrane staining), ‘2’ (medium membrane staining), and ‘3’ (high membrane staining). HercepTest was classified according to the standard procedures and translated to our classification system as follows: HercepTest score ‘0’ and ‘1+’ as ‘1’ (negative for *Her2/neu* expression), score ‘2+’ as ‘2’ (weak positive), and score ‘3+’ as ‘3’ (strong positive). Results of both independent interpretations (already the average of the three biopsies per patient on the microarrays) were averaged and newly classified (1.00–1.66=‘1’, 1.67–2.33=‘2’, and 2.34–3.00=‘3’). Data were analysed statistically with SPSS 13 (SPSS, Chicago, IL, USA). *P*-values below 0.05 were considered statistically significant. *P*-values above 0.1 were signed simply as NS (not significant). *P*-values in between were signed NS, with the corresponding *P*-value in parentheses. Correlations among clinicopathologic parameters were calculated using the Pearson's *χ*^2^ or Fisher's exact test as appropriate and were corrected for multiple testing (Bonferroni–Holmes). Correlation of staining intensities of the putative oncoproteins among each other and with International Federation of Gynecology and Obstetrics (FIGO) stages was calculated using the Spearman's Correlation Test and corrected for multiple testing (Bonferroni–Holmes).

Univariate Cox models were used to demonstrate the influence of known prognostic factors and the four potential new prognostic factors. For each of the new factors, a multiple Cox model with known prognostic factors as adjustment variables was calculated.

## RESULTS

### Description of patient cohort

Clinical and histopathological characteristics of patients included in this study show a typical ovarian cancer population and are presented in Table 1. Mean age of patients at first diagnosis was 58.6 years (range 27.6–87.2 years). 54.7 of patients with malignant tumours had serous adenocarcinomas and 62.5% had stage III/IV disease. Of the 128 patients with malignant tumours, 81 patients (67.5%) received carboplatin–paclitaxel-based standard chemotherapy, nine patients (7.5%) received cisplatin–cyclophosphamid-based chemotherapy, 14 patients (11.7%) another regimen, and 16 patients (13.3%) no systemic therapy at all. For eight patients, no information about systemic treatment was available.

Median follow-up for patients with malignant tumours was 43.7 months (range 0.4–168.7 months), and 39 patients (26.4%) had already died. None of the patients with borderline tumours died during the follow-up time of median 45.7 months (range 0.6–120.9 months).

### HER2/neu overexpression in ovarian cancer samples

*HER2/neu* protein was stained with the DAKO HercepTest and interpreted following the standard procedures for breast cancer diagnosis. 35 out of 127 cancer tissues (27.6%) of patients with malignant tumours were found to overexpress the *HER2/neu* gene product including four tissues with high *HER2/neu* expression (3+) (Table 2). Only three out of 20 tissues (15.0%) of patients with borderline tumours showed overexpression of *HER2/neu*, none of them with high expression. Table 2 shows the prevalence of tumour staining scores with respect to histology, FIGO stage, and grade. There was no difference between *HER2/neu* staining regarding these clinical characteristics.

### CXCR4 expression in ovarian cancer samples

**Figure 1:**
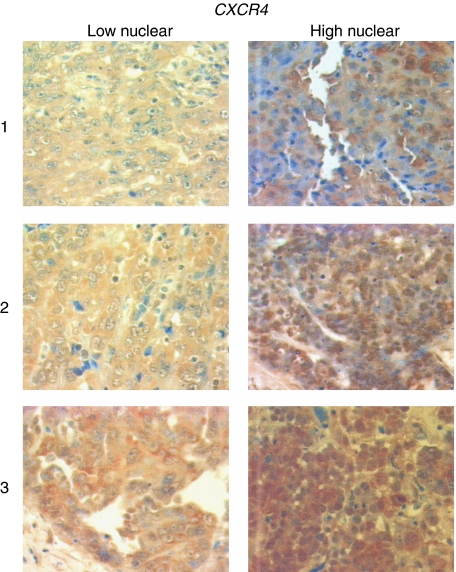
Immunohistochemistry staining of *CXCR4* on different malignant ovarian cancer tissues. In the left-hand panel, representative tissues with low nuclear and low (1), medium (2), or high (3) cytoplasmic *CXCR4* stainings are shown. In the right-hand panel, representative tissues with high nuclear and low (1), medium (2), or high (3) cytoplasmic *CXCR4* stainings are shown.

We examined expression of *CXCR4* in the same tissues as described above. *CXCR4* expression was found independently in the cytoplasm and/or in the nucleus (Salvucci *et al*, 2005). In positive cases, membrane staining of *CXCR4* was not distinguishable from high cytoplasmic staining. This is in accordance with similar experiments with breast cancer tissues and the same primary antibody (Salvucci *et al*, 2005). Cytoplasmatic staining was classified as 1 (missing or weak expression), 2 (medium expression), and 3 (high expression). Nuclear expression was classified as ‘Yes’ (visible staining) and ‘No’ (no visible staining of the nucleus) (Figure 1, Table 2). The relatively low standard deviations within the three corresponding tumour cores from different positions of the tumour on the tissue microarray (the mean of the standard deviations over all patients equals 0.22) points to a low variability of cytoplasmic *CXCR4* expression within one tumour. In 53.8% of malignant tumours, cytoplasmatic *CXCR4* expression was medium or high (2/3) and 21.8% showed *CXCR4* staining of the nucleus. There was no correlation of *CXCR4* staining in the cytoplasm and the nucleus (Table 3). No significant different expression of nuclear and cytoplasmic staining regarding, histology, FIGO stage, or grade could be found; only the nuclear expression appeared indirectly correlated to grading (but not significant after correction for multiple testing), starting at 37.5% positive tumours for grade 1, 30.0% positive tumours for grade 2, and ending with 12.7% positive tumours for grade 3 (Table 2). Neither the cytoplasmatic nor the nuclear *CXCR4* expression was correlated with the *HER2/neu* expression in ovarian cancer tissues (data not shown). *CXCR4* stained high or medium in the cytoplasm of 50% borderline tumours and in 33.3% of borderline tumour nuclei, which was not different from the staining frequencies in malignant tumours (Table 2).

### SDF-1 abundance in ovarian cancer tissues and stroma

To get some insight into the functionality of *CXCR4* receptors on the surface of ovarian cancer cells, we included an analysis of *SDF-1* on the cell membrane – the only known soluble ligand of *CXCR4*.

**Figure 2:**
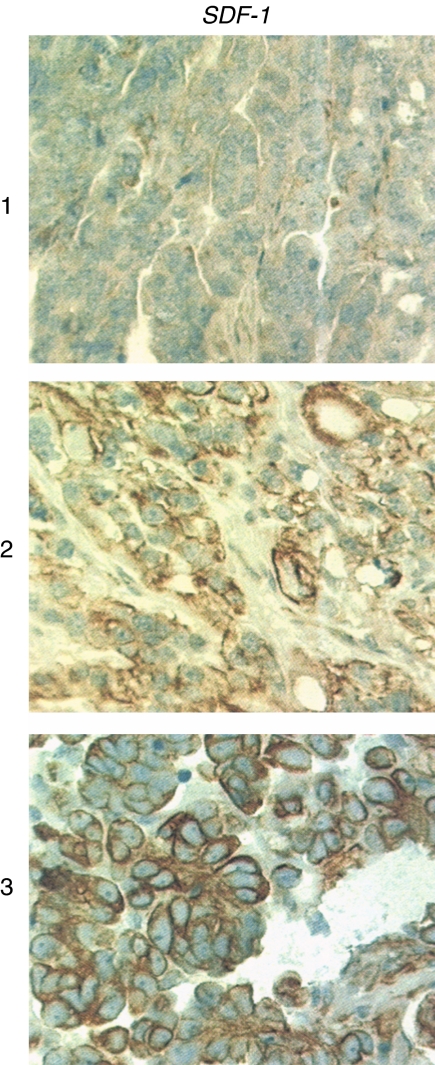
Immunohistochemistry staining of *SDF-1* (*CXCL12*) of different malignant ovarian cancer tissues. The representative stainings of tissues with low (virtually missing) (1), medium (2), and high (3) *SDF-1* abundance are shown. Notice prevalent *SDF-1* staining of endothelium in the right upper corner of the middle picture.

For *SDF-1*, 32.0% of membranes of malignant tumour samples stained medium or high (2/3) (Figure 2). Positive staining did not correlate with any clinicopathologic characteristics like histology, FIGO stage, or grade (Table 2). Membranous *SDF-1* staining correlated significantly with the cytoplasmatic *CXCR4* expression, as expected (correlation *r*=0.373, *P*< 0.001; Table 3), but, interestingly, – indirectly, also as a trend with the nuclear *CXCR4* expression (Spearman correlation rr=−0.244; *P*=0.084 after correction; Table 3). No correlation of *SDF-1* abundance and *HER2/neu* expression was found (data not shown). Medium or high (2/3) *SDF-1* protein levels were found in 31.5% of membranes of borderline tumours, which is the same frequency as for malignant tumour samples. As expected, *SDF-1* was expressed at low level in the stroma of all ovarian cancer cases (data not shown).

### Overall survival analysis

**Figure 3:**
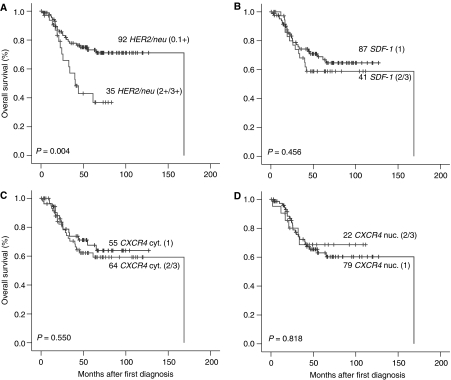
Plots of Kaplan–Meier estimates for overall survival of patients with tumour tissues with low (1) or high (2/3) *HER2/neu* (**A**), low (1) or high (2/3) *SDF-1* (**B**), low (1) or high (2/3) cytoplasmic *CXCR4* (**C**), and low (1) or high (2/3) nuclear *CXCR4* (**D**) expression/abundance. *P*-values are from the log-rank test.

For Kaplan–Meier plots with patients with malignant tumours, the parameters were dichotomised in two groups, one with low (1) *HER2/neu* or cytoplasmic *CXCR4* expression or *SDF-1* abundance and one with medium or high (2/3) expression/abundance of the corresponding protein (Figure 3). The only parameter with significant univariate influence on prognosis was *HER2/neu* (*P*=0.004; Figure 3A). The medium overall survival for patients with *HER2/neu* overexpression was 40.3 months compared with 168.7 months for the *HER2/neu*-negative patients. Type of systemic chemotherapy (carboplatin–paclitaxel based *vs* other) had no significant influence on the prognostic value of the *HER2/neu* overexpression status (data not shown).

All other parameters – cytoplasmic or nuclear *CXCR4* expression and *SDF-1* abundance – had no influence on overall survival. The 75% percentile of overall survival for all groups with low or high expression of each of these three parameters was very similar and ranged from 28.6 to 33.4 months (Figure 3B–D). There was no subgroup, for example, histological, FIGO stage, or grade, which resulted in a significant influence of these three parameters under investigation on patient prognosis (data not shown). There was also no combination of variables, for example, only *CXCR4* and *SFD-1*-positive tumours compared with others, which resulted in a significant influence on patient prognosis (data not shown). Relative risk of patients with positive *CXCR4* expressing tumours but negative staining for *SDF-1* was not significantly higher compared with patients with negative *CXCR4* expressing tumours in the same background (relative risk of 1.57, *P*=0.281).

A multiple analysis revealed FIGO stage and grade as the only independent prognostic factors for overall survival in our cohort of 128 malignant ovarian cancer patients. Of all parameters under investigation, only *HER2/neu* showed negative trend, also, for overall survival (relative risk of 1.92, *P*=0.074; Table 4) after multiple corrections also a negative trend also for overall survival (relative risk of 1.92, *P*=0.074; Table 4).

A multiple analyses of the impact of *HER2/neu* expression on overall survival, using both *CXCR4* expressions (nuclear and cytoplasmic) and the *SDF-1* abundance as correcting variables showed that there was no influence of one of these three parameters on the prognostic value of *HER2/neu* expression (data not shown). Survival analysis was performed only with patients of malignant tumours, because patients with borderline tumours had a significant better prognosis, with no cases of death during follow-up.

## DISCUSSION

Hereditary ovarian and breast cancers are based on germline mutations in the same cancer susceptibility genes, *BRCA1* and *BRCA2*, suggesting similar pathways of oncogenesis at least for a fraction of these diseases. Moreover, both cancer types show, in a comparable percentage, overexpression of the oncoprotein *HER2/neu.*

In breast cancer, the diagnostic and therapeutic possibilities of *HER2/neu* expression are well explored. Well known since decades as an adverse prognostic factor, the more recent insight that breast cancers expressing *HER2/neu* are more susceptible to anthracycline-based chemotherapy (Pritchard *et al*, 2006) as well as the introduction of the *HER2/neu* antibody trastuzumab into the adjuvant setting has had significant impact on the prognosis of this particular subgroup of breast cancer patients (Slamon *et al*, 2001).

In ovarian cancer, the prognostic influence of *HER2/neu* is still a matter of debate and the therapeutic capacity of the available drugs to target the *HER2/neu* pathway are insufficiently explored. Even the percentage of *HER2/neu*-positive patients varies considerably among individual studies. The lack of knowledge about the prognostic and therapeutic impact of *HER2/neu* expression in ovarian cancer may be partly explained by its lower prevalence in the general population resulting in slower patient recruitment and underpowered studies. Besides this simplistic view, the possibility of a less significant and/or different influence of *HER2/neu* expression in breast and ovarian cancer could be another and more challenging explanation.

Even in breast cancer where the functionality of *HER2/neu* has been extensively studied, its role in oncogenesis is still far from being understood. Recently, interesting functional data were generated, showing that *HER2/neu* enhances expression of the chemokine receptor *CXCR4*. *CXCR4* is furthermore crucial for *HER2/neu* induced invasion, migration, and adhesion activities, and *in vitro HER2/neu* protects *CXCR4* from ligand-induced protein degradation. *CXCR4* is furthermore responsible for *HER2/neu*-induced lung metastases *in vivo*. These *in vivo* findings are corroborated by the observation that *CXCR4* is upregulated in *HER2/neu* overexpressing primary breast tumour tissues and is correlated with poor patient survival (Li *et al*, 2004). The findings that cytoplasmic *CXCR4* expression is elevated in breast cancer samples, and that higher expression of *CXCR4* is associated with parameters of tumour aggressiveness, and a poor prognosis was later confirmed in an independent patient population (Salvucci *et al*, 2005).

In accordance with the majority of publications, we found *HER2/neu* to be overexpressed in about a quarter of malignant tumours. *HER2/neu*-positive patients had a significantly shorter overall survival. There was a trend that *HER2/neu*-positive patients were diagnosed with a higher FIGO stage, resulting in the fact that in a multivariate model *HER2/neu* positivity did not hold as an independent variable. *HER2/neu*-positive tumours did not show a higher expression of cytoplasmic *CXCR4* staining, which was positive in over half of the cases and correlated closely with the expression of its ligand *SDF-1* as expected. There was no impact of cytoplasmic and nuclear *CXCR4* expression or *SDF-1* abundance on overall survival (Figure 3 and Table 4).

This is in contrast to breast cancer and also other forms of cancer where *CXCR4* has been shown to be of significant prognostic influence like oesophageal cancer (Kaifi *et al*, 2005), colon cancer (Kim *et al*, 2005), early-stage non-small-cell lung cancer (Spano *et al*, 2004), low-grade glioma (Salmaggi *et al*, 2005), malignant melanoma (Scala *et al*, 2005), osteosarcoma (Laverdiere *et al*, 2005), oral squamous cell carcinoma (Almofti *et al*, 2004), and adult acute myeloid leukaemia (Rombouts *et al*, 2004). One explanation might be the importance of distant metastases to *SDF-1* expressing tissues on the cause of death in most of these other forms of cancer. Patients only rarely die of their primary cancer, but rather as a result of metastatic disease. In ovarian cancer, recurrences in the pelvis and, in most cases even within the peritoneum, which has a comparable microenvironment as the primary tumour, are the main causes for death. In our series, only 6.3% (eight out of 128) of patients had distant metastases at primary diagnosis (FIGO 4) and therefore a statistical analysis for *CXCR4* overexpression from this group was fruitless. No difference in *HER2/neu*, *CXCR4*, and *SDF1* expression (or combination) could be found in this small subgroup. Most patients with ovarian cancer die on local recurrences (transcoelomic metastases) within the peritoneum and not on distant metastases (haematogenous metastases) (Vaccarello *et al*, 1995). In fact, only about 15% of patients get distant metastases during their course of disease (Sood *et al*, 1999). Further support came from the more recent finding that intraperitoneal chemotherapy is equivalent and even superior to systemic therapy in this disease (Armstrong *et al*, 2006). Thus, the biology of ovarian cancer seems to be quite different from other epithelial cancers (Naora and Montell, 2005). As an example, normal cells of the ovarian surface epithelium express only little or even no E-cadherin and have both mesenchymal and epithelial features, whereas many primary ovarian carcinomas express higher levels of E-cadherin. The gain of E-cadherin expression, completely unexpected for tumour cells, may result in an advantage for ovarian cancer cells to colonise new sites in the peritoneum. The characteristic epithelia–mesenchymal transition — thought to be necessary for distant metastases, – as well as for the development of distant (haematogenous) metastases, seems to play a subordinate role in the course of ovarian cancer disease. Thus, our finding of the missing influence of *CXCR4* expression (and *SDF-1* abundance) on patient outcome is in line with the above-mentioned fact that haematogenetic metastases to organs with high *SDF-1* expression is relatively rare in ovarian cancer. Molecular mechanisms for these differences are not well understood in detail (Tan *et al*, 2006) but are in the focus of increasing scientific endeavours.

In summary, no clear-cut relationship between *HER2/neu, CXCR4*, *SDF1*, and metastasis and/or prognosis as obvious for breast cancer was found in ovarian cancer. If *HER2/neu* expression is of biological relevance and not merely reflecting a more advanced stage of the disease, other than *CXCR4*-mediated *HER2/neu* activities have to be explored for ovarian cancer.

## Figures and Tables

**Table 1:** Clinicopathologic characteristics of patients

**Ovarian tumours**	***n*=148**	**Mean±s.d.**
*Age (years)*		
Borderline	20	54.7±13.9
Malignant	128	59.2±12.1
		
*Histology*		
Borderline	20	13.5%
Serous	70	47.3%
Endometrioid	24	16.2%
Mucinous	7	4.7%
Clear cell	7	4.7%
Mixed	8	5.4%
Undifferentiated	12	8.1%
		
*FIGO stage*		
1	51 (17 borderline)	34.5%
2	14	9.5%
3	75 (3 borderline)	50.7%
4	8	5.4%
		
*Grade*		
Borderline	20	13.5%
G1	21	14.2%
G2	26	17.6%
G3	70	47.3%
Missing	11	7.4%
		
*Follow up (months)*	Median	Range
Borderline	45.7	0.6–120.9
Malignant	43.7	0.4–168.7

Abbreviations: FIGO=International Federation of Gynecology and Obstetrics; s.d.=standard deviation.

**Table 2:** Correlation of *HER2/neu*, cytoplasmic or nuclear *CXCR4* expression, and *SDF-1* abundance with clinicopathologic characteristics

	**HER2/neu**					**CXCR4**							**SDF-1**				
						**Cytoplasmic**					**Nuclear**						
	**1** a	**2**	**3**	***N***	***P***	**1**	**2**	**3**	***N***	***P***	**Yes/no**	***P***	**1**	**2**	**3**	***N***	***P***
*Malignity*					NS^b^					NS^b^		NS^c^					NS^b^
Borderline	17	3	0	20		9	8	1	15		5/15		12	6	1	19	
Malignant	92	31	4	127		55	51	13	119		22/101		87	30	11	128	
																	
*Histology (malignant)*					NS^b^					NS^b^		NS^b^					NS^b^
Serous	52	14	3	69		25	34	6	65		10/55		46	17	7	70	
Endometrioid	18	5	1	24		10	9	4	23		5/20		15	6	3	24	
Mucinous	6	1	0	7		3	3	0	6		4/6		6	1	0	7	
Clear cell	5	2	0	7		5	1	0	6		1/5		6	1	0	7	
Mixed	5	3	0	8		4	2	1	7		1/4		5	2	1	8	
Undifferentiated	6	6	0	12		8	2	2	12		1/11		9	3	0	12	
																	
*FIGO stage*					NS												
					NS (0.064)^d^					NS^d^		NS^d^					NS^d^
1	42	9	0	51		18	23	5	46		11/41		33	11	6	50	
2	12	2	0	14		8	4	0	12		2/10		9	5	0	14	
3	51	20	3	74		33	30	8	71		14/58		53	17	5	75	
4	4	3	1	8		5	2	1	8		0/7		4	3	1	8	
																	
*Grade (w/o borderline)*					NS^b^					NS^b^		NS^b^					NS^b^
G1	18	3	0	21		6	9	3	18		6/16		12	7	2	21	
G2	18	7	1	26		12	11	2	25		6/20		20	3	3	26	
G3	46	21	2	69		31	28	7	66		7/55		47	19	4	70	

a For consistency with our rating system, *HER2/neu* scores were translated as follows: ‘0’ and ‘1+’=‘1’, ‘2+’=‘2’, and ‘3+’=‘3’.

b Pearson's *χ*^2^ test (corrected).

c Fisher's exact test (corrected).

d Spearman's correlation test, corrected for multiple testing.

Abbreviations: NS=not significant, FIGO=International Federation of Gynecology and Obstetrics.

**Table 3:** Correlation of *SDF-1* abundance and cytoplasmic or nuclear *CXCR4* expression

	**CXCR4 cyt.**					**CXCR4 nuc.**	
*Borderline*							
	NS^a^	**1**	**2**	**3**	NS^a^	**No**	**Yes**
SDF-1	**1**	7	3	0	**1**	5	2
	**2**	2	4	0	**2**	3	3
	**3**	0	1	0	**3**	1	0
							
*Malignant*							
	<0.001^a^	**1**	**2**	**3**	NS (0.084)^a^	**No**	**Yes**
SDF-1	**1**	45	31	3	**1**	47	19
	**2**	9	13	7	**2**	22	3
	**3**	1	7	3	**3**	10	0
	NS^a^						
CXCR4 nuc.	**No**	36	33	10			
	**Yes**	12	8	2			
							

a Spearman's correlation test (corrected). NS, not significant.

**Table 4:** Hazard ratios for overall survival of the 128 patients with malignant tumors after multiple Cox regression analyses of *HER2/neu* overexpression, *SDF-1* abundance, and cytoplasmic or nuclear *CXCR4* expression

	***HER2/neu* (0/1+ *vs* 2+/3+)**			***SDF-1* (1 *vs* 2/3)**			***CXCR4* cyt. (1 *vs* 2/3)**			***CXCR4* nuc. (No *vs* Yes)**		
	**HR^a^**	**95% CI^b^**	***P***	**HR**	**95% CI**	***P***	**HR**	**95% CI**	***P***	**HR**	**95% CI**	***P***
*Histology*												
Serous	1.00			1.00			1.00			1.00		
Non-serous	1.06	0.50–2.22	0.875	1.24	0.60–2.53	0.559	1.34	0.65–2.78	0.434	1.10	0.48–2.52	0.819
												
*FIGO stage*												
I+II	1.00			1.00			1.00			1.00		
III+IV	15.38	1.96–125.00	0.009	19.61	2.54–142.86	0.004	18.18	2.37–142.86	0.005	10.75	1.34–83.33	0.025
												
*Grade*												
G1+G2	1.00			1.00			1.00			1.00		
G3	3.47	1.19–10.10	0.022	3.60	1.24–10.42	0.019	3.32	1.15–9.62	0.027	6.21	1.38–27.78	0.017
												
*HER2/neu*												
0/1+	1.00											
2+/3+	1.92	0.94–3.94	0.074	NA^c^			NA			NA		
												
*SDF-1*												
1				1.00								
2+3	NA			1.36	0.68–2.75	0.385	NA			NA		
												
*CXCR4 cyt.*												
1							1.00					
2+3	NA			NA			1.37	0.68–2.76	0.379	NA		
												
*CXCR4 nuc.*												
No										1.00		
Yes	NA			NA			NA			1.51	0.55–4.08	0.421
												

a Hazard ratio.

b 95% confidence interval.

c Not applicable.

Abbreviations: FIGO=International Federation of Gynecology and Obstetrics.
